# Machine Learning Against Terrorism: How Big Data Collection and Analysis Influences the Privacy-Security Dilemma

**DOI:** 10.1007/s11948-020-00254-w

**Published:** 2020-07-21

**Authors:** H. M. Verhelst, A. W. Stannat, G. Mecacci

**Affiliations:** 1 Delft Institute of Applied Mathematics, Delft University of Technology, Van Mourik Broekmanweg 6, 2628XE Delft, The Netherlands; 2 Donders Institute for Brain, Cognition and Behaviour, Radboud University, Montessorilaan 3, 6525 HR Nijmegen, The Netherlands

**Keywords:** Privacy-security dilemma, Mass surveillance, Metadata collection, Machine learning, National security

## Abstract

Rapid advancements in machine learning techniques allow mass surveillance to be applied on larger scales and utilize more and more personal data. These developments demand reconsideration of the privacy-security dilemma, which describes the tradeoffs between national security interests and individual privacy concerns. By investigating mass surveillance techniques that use bulk data collection and machine learning algorithms, we show why these methods are unlikely to pinpoint terrorists in order to prevent attacks. The diverse characteristics of terrorist attacks—especially when considering lone-wolf terrorism—lead to irregular and isolated (digital) footprints. The irregularity of data affects the accuracy of machine learning algorithms and the mass surveillance that depends on them which can be explained by three kinds of known problems encountered in machine learning theory: *class imbalance*, *the curse of dimensionality*, and *spurious correlations*. Proponents of mass surveillance often invoke the distinction between collecting data and metadata, in which the latter is understood as a lesser breach of privacy. Their arguments commonly overlook the ambiguity in the definitions of data and metadata and ignore the ability of machine learning techniques to infer the former from the latter. Given the sparsity of datasets used for machine learning in counterterrorism and the privacy risks attendant with bulk data collection, policymakers and other relevant stakeholders should critically re-evaluate the likelihood of success of the algorithms and the collection of data on which they depend.

## Introduction

In the past decades, governments around the world have increased their use of automated intelligence for the collection and evaluation of data in efforts to ensure national security. The PRISM programme run by the US National Security Administration (NSA) became one of the best known examples of large scale wiretapping operations after the leaks by Edward Snowden in 2013. Yet there are many other ‘upstreaming’ programmes (i.e., direct tapping into communications infrastructure for data interception) that are used by governments, including the United Kingdom, Germany, Sweden, France, and The Netherlands. Their goal is to detect suspicious behaviour of individuals within a large group of citizens (Bigo et al. 2013).

Snowden’s disclosures revealed that the NSA had wiretapped millions of American citizens and had collected copious phone and email records. This surveillance method, often referred to as *wiretapping* or *dragnet surveillance*, outraged many Americans, who considered it a violation of their privacy. However, the surveillance tactics were frequently defended on the grounds that the collection of data had been confined to *metadata* (i.e., data about data), not actual data, and so it was less intrusive (a claim that we challenge in the third section). Opposition to these tactics—in contrast to general acceptance of targeted surveillance of individuals who have aroused suspicion—can be explained by the *privacy*-*security dilemma* (Van den Hoven et al. 2012). The *privacy*-*security dilemma* describes the trade-off between the people’s right to privacy and their right to security, whereby the challenge lies in finding a reasonable balance between the two.^1^

The rapid improvement of information and communication technologies facilitates the collection and analysis of increasing amounts of data in shorter periods of time, making dragnet surveillance a viable and appealing alternative to targeted surveillance. Improvements in computing power have resulted in the increased performance of machine learning algorithms in recent years. By utilising large data sets, machine learning has been shown to be able to recognise objects in pictures, e.g., detecting breast cancer (Liu et al. 2017); listen and respond to speech commands as Apple’s Siri or the Google Assistant do; and defeat the world champion in the game Go (Google’s AlphaGo) (Borowiec 2016). The increased performance of machine learning algorithms on complex problems has changed the balance of the privacy-security dilemma with respect to dragnet surveillance, necessitating a re-evaluation of the relation of effectiveness to intrusiveness of the techniques employed.

In this paper, we aim to assist policymakers in achieving a clearer understanding of the challenges in machine learning for counterterror. This is meant to aid their decision making when faced with the privacy-security dilemma in the contemporary context of machine learning algorithms and bulk data collection. We first consider the performance of machine learning algorithms for national security and argue that these algorithms are likely improperly trained due to three kinds of well-known problems in machine learning research—class imbalance, curse of dimensionality, and spurious correlations. Then we consider the relation between machine learning algorithms and metadata collection in their ability to reveal seemingly anonymised information. Based on this analysis, we conclude that the characteristics of the datasets typically utilised in counterterrorism make it unlikely that machine learning algorithms will significantly increase national security, and the privacy concerns that arise through the bulk data collection necessary for these projects—the use of metadata in particular—requires reconsideration of the privacy-security trade-off.

## Machine Learning for Mass Surveillance

Machine learning, a branch of artificial intelligence, is one of the fastest-growing areas of computer science, with far-reaching applications in many fields. The term refers to the automated detection of patterns and recurrences in large data sets through various “training” techniques. Machine learning algorithms are “fed” artificial data sets (training sets) containing patterns from which they are meant to learn to detect such patterns in real-life data sets. When applied to mass surveillance, the training sets consist of personal data or features of a person (e.g., name, year of birth, contents of emails, phone calls, etc.) and a label set that indicates whether intelligence agencies consider the person a threat. The trained algorithm is then applied to an unlabelled data set with the same features, and it labels individuals as “threats” based on what it has learned. The accuracy of these algorithms depends on the characteristics of the data set and the amount of data that is used for training.

Despite the lively ethical debate (Brayne 2017), applications of machine learning algorithms for policing are increasingly common. One example is for recognising particular areas as hot spots for crime. Matijosaitiene et al. (2019) have achieved very high accuracy in predicting car theft in urban areas of New York City. Camacho-Collados and Liberatore (2015) developed a decision support system that proposes when and where police patrols should be deployed based on data sets that capture the time and place crimes were committed in the past. These algorithms are used to identify where and when crimes are likely to be committed, but do not identify who will commit crimes, i.e. mark individuals as potential criminals.

The data available for the enforcement of counterterrorism measures through machine learning algorithms is different from the data that is used in policing. Terror attacks, especially those committed by lone actors unaffiliated with any organisation, are highly diverse in their motives, planning and execution (Jonas and Harper 2006; Lindekilde et al. 2019). This implies that digital footprints of (potential) terrorists can vary significantly, which leads to isolated points in the training data. This makes the training of machine learning algorithms more difficult as the uniqueness of many attacks increases the probability of inadequate training and consequently inaccurate algorithms. A common approach to solving the problem of irregular data points is to increase the size of the training dataset. As more training data naturally implies a higher probability of achieving more reliable and accurate algorithms, the irregularity of data points can be amended in this way. In the case of localized crime prediction this can be very successful, whereby the set of data about crime used to train the algorithm is simply increased to a size that returns adequately accurate results (Matijosaitiene et al. 2019). However, unlike in the case of predicting mundane crime, datasets related to (potential) terrorist attacks cannot be expanded so easily. This is due to the fact that labelling datasets requires (potential) terrorists to be already identified as a threat which is difficult for authorities to reliably do. Although the United States Department of Homeland Security has assembled an extensive watch list over the past decade (which per se might give the impression that available training data should be sufficient to achieve accurate results), the lists they produce have been proven to be quite inaccurate, containing large number of false positives, i.e., people wrongly labelled as threats (Soghoian 2008). Expanding the training data from actual terrorists to also include those identified on these lists, although prima facie appealing to solve the problem that arises with a small dataset, would likely imply that algorithms inherit bias from the data set and produce large numbers of falsely identified threats.

The challenges that these currently available counterterrorism datasets create in training machine learning algorithms are explained by the three well known mathematical phenomena of class imbalance, curse of dimensionality and spurious correlations (L’Heureux et al. 2017).

Since terrorist attacks are distinct in their motives, planning, and execution, the digital footprints left behind by terrorists during the preparation of attacks vary based on the impetus for their actions (e.g., religious or political convictions or mental health issues), the number of people involved, and the ways they communicated (which can be encrypted or offline) (Sirseloudi 2005). Additionally, clues might be hard to find in one single data set, but could become more clear when combining data from different sets. These characteristics require a large number of features or details to be analyzed and yield a high ‘dimension’ of the domain set. Ultimately, machine learning algorithms for mass surveillance should be trained on a large number of different data sets in order to be able to find a relation between those. This challenge of machine learning for big data is called *the curse of dimensionality,* which means that the complexity of statistical inference, which grows with the dimension of the data set, degrades the accuracy and performance of the machine learning algorithm (L’Heureux et al. 2017). For mass surveillance, this implies that selecting more details per suspect can increase the accuracy of the algorithm, but entails a need for a much more extensive data set. Hence more data points of terrorist attacks are required, which simply means that the number of recorded terrorist attacks might be too small for proper training.

*Class imbalance* denotes the “non-uniformity” of the training data set (L’Heureux et al. 2017). That is, when a machine learning algorithm is trained to identify whether a person is more likely to commit a terror attack, the data set used for training should consist in equal proportions of positives and negatives, i.e., terrorist and non-terrorists. Although machine learning methods are reported to work for slightly imbalanced data sets, a data set representing the population of a state, country, or region has nowhere close to a balance of terrorists and non-terrorists. As a consequence, in order to balance the data, in this case, training is done using uniformized subsets (or “parts”) of the training sets. This means that, to maximise accuracy of the algorithms, training subsets should either be reduced in terms of dimensions or ‘features’, sacrificing robustness against the variability in the footprint of unsuspicious individuals, or the training subsets should—somehow—be enriched with the footprints of (potential) terrorists, which is challenging as mentioned before. The fact that (potential) terrorist attacks are different in their motives, planning and execution can impose difficulties since class imbalance is more likely to occur with datasets of a high dimension. If balancing or uniformization of training sets is not possible, a relatively small number of subdomains can be made, which leads to an increased risk of *overfitting*, i.e., performance that is satisfactory on a training set but unsatisfactory on real data. When training sets are hard to balance, proper training of the algorithm is questionable, and the accuracy of the algorithm is uncertain.

A third issue comes as a consequence of dealing with *spurious correlations* (L’Heureux et al. 2017). In big data analysis, it is known that adding more features to a data set increases the probability of finding correlations in the data, of which some are meaningless. For example, one might find a correlation between the probability of an individual being a terrorist and their shoe size. These correlations can arise regardless of whether the data set is balanced, and thus are rather difficult to prevent. They are found between two features that are not necessarily causally related and often cause *overfitting* of the algorithm. One should be aware that the typical high-dimensionality of data sets used for national security makes spurious correlations and overfitting more likely to occur (Calude and Longo 2017).

The aforementioned challenges tend to significantly reduce machine learning algorithms’ classification accuracy. When applied to mass surveillance, this might lead to outputs that contain a high degree of uncertainty, leading to misclassifications of the threat level of both terrorists and innocent citizens. If a ML method is inaccurate, it might lead to overestimations or underestimations of the level of threat. In order to correct this, other methods of surveillance will need to be used to complement it, together with a significant effort in terms of human supervision. A large monitored population implies that even a highly accurate algorithm can lead to a large number of incorrect evaluations for which human supervision (i.e., human intelligence) or cross checking with different methods will be needed. As an illustrative example, an algorithm with a relatively high accuracy of 99.9%, applied to a data set of a population of 10 million people, will offer inaccurate evaluations of 10,000 people. Increasing the population or decreasing the algorithmic accuracy increases the need for supervision. This problem is even more substantial when we consider that mathematical estimates of the general performance, and more importantly of the accuracy of machine learning algorithms, have not yet been developed. While the judgment of the accuracy of a machine learning algorithm on the labelled training set is possible, its performance in ‘the real world’ cannot be estimated a priori (Begoli et al. 2019; Dunson 2018). This means that, in case of *overfitting* of the algorithm on the training set, the method’s accuracy—*on the training set*—is often found to be relatively high, whereas the accuracy *on the total dataset* is often significantly lower. Hence, when evaluating machine learning methods and the performance of mass surveillance techniques, policymakers and other non-expert stakeholders should be aware that the accuracy of an algorithm on the training set might not correctly represent its accuracy in real-world scenarios, i.e., when applied to full populations. Formal mathematical indicators (i.e. uncertainty quantification Dunson 2018) that predict algorithmic performance on full populations are currently an open topic of research and cannot be relied upon yet.

In the context of the privacy-security dilemma, when methods turn out to be inaccurate, the privacy of many individuals is sacrificed while gains in national security are limited. We suggest that theoretical challenges inherent to machine learning techniques, i.e. *class imbalance, the curse of dimensionality and spurious correlations*, should be considered in determining, case by case, the likely and actual efficacy of national security strategies. We also recommend to carefully prioritize methods in which uncertainty levels can be best quantified, in order to form correct expectations on what the accuracy of a technology could be when applied to big datasets and real-world scenarios.

## The Use of Metadata with Machine Learning

So far, we have established that machine learning techniques applied to mass surveillance suffer from inherent performance limitations, which reduce their enhancement of security, and that they constitute a rather large invasion of privacy. Keeping in mind the privacy-security dilemma one could increase the overall justifiability of these methods by rendering them less invasive. A way of doing this is by confining the data analysed to *metadata* (i.e. ‘data about data’), which is one of the main arguments proposed by advocates of mass surveillance in order to mitigate privacy concerns (Kift and Nissenbaum 2016). For example, for email and phone records, data is the content of the email or call, while metadata includes the length and time of communication and the phone numbers or email addresses of those communicating, but not what was written or said. Since metadata does not contain the data itself, it is generally regarded as a less invasive source of information than actual data. This *appeal to metadata* however, is at least partially flawed (Landau 2013). In justifying this, we consider two factors: first, the strengths of machine learning algorithms with respect to pattern recognition and their ability to reveal sensitive content; second, the rather “liberal” use of the term *metadata*. These aspects, discussed in the following paragraph, might make the mentioned *appeal to metadata* harder to justify. However, objections against the mining of *metadata* for mass surveillance have been raised on the basis of it being more severely privacy breaching than initially claimed (Naughton 2013; Schneier 2015).

It has been shown that machine-learning algorithms are able to infer much more sensitive information from the acquired metadata, allowing for a much deeper look into people’s lives. For example, using credit card data containing the features time, place and price range of purchases made, De Montjoye et al. (2015) successfully identified the owners of anonymous credit cards. In another case, the same authors were able to identify location and movements of mobile phones using anonymised phone carrier antenna data based on small numbers of antenna communications (de Montjoye et al. 2013). Another example is that of telephone metadata (Mayer et al. 2016). Solely based on cell-phone activity metadata (i.e., which numbers were called at what times) machine learning algorithms were able to infer personal information about the owners of the phones. For instance, whether or not a person owns a firearm and even what types of health issues they might have. Furthermore, Narayanan and Shmatikov (2008) used the anonymous dataset of Netflix movie ratings to train an algorithm that is able to find which ratings of the set belong to the same user. These examples show that in the last decade, automated algorithms have been able to de-anonymise datasets in several applications, uncovering private information. As algorithms are expected to become more advanced over time, the collection of metadata should no longer be seen as just a minor infringement of people’s privacy as de-anonymisation of large metadata sets with more features or more records will be even faster.

Besides the revelations of metadata, Feigenbaum and Koenig (2014) note that there is no rigorously defined distinction between metadata and data. As an example, they mention that the metadata of an email client comprises IP addresses of the sender and the receiver. The IP address, however, is considered data from the perspective of an access point or a router. This shows that an IP address is considered metadata by one system (email) but data by the other (internet trafficking). Since the distinction between metadata and data is not clear in all cases, a solid legal framework about what (meta)data can be collected and what not, is essential to prevent loopholes in data collection by intelligence agencies and their partners.

In conclusion, we remind the reader that the impact of confining data collection to metadata on the breach of personal privacy might be underestimated. Considering massive metadata collection and analysis for counterterrorism, personal details about many individuals occurring in the dataset can be revealed using machine learning, as has been shown in the past decade by several studies. We advise policymakers and other relevant stakeholders to consider the actual costs in terms of privacy that such methods, even when confined to metadata analysis, will likely entail; a problem that is likely only to get worse.

## Conclusion

When using a data set with a sufficient number of labelled cases, machine learning is able to assist law enforcement in detecting localised criminality, thus answering the question where and when crimes will be committed. However, when answering questions about who will commit a crime, in particular a terror attack, the features of datasets increase and data points are more distinct, leading to machine learning algorithms that are potentially less accurate. Well-known machine learning problems of class imbalance, the curse of dimensionality and spurious correlations lead us to believe there will be inaccuracies due to *over*- *or underfitting*. Consequently, the number of false positives and false negatives is expected to be very high when using these algorithms in enforcing national security, and general a priori uncertainty estimates of machine learning algorithms for the full population rather than the training set are currently missing.

Furthermore, the use of metadata instead of data does not necessarily decrease the breaches of an individual’s privacy. Using studies from literature from the past decade, which all focussed on de-anonymisation of datasets on phone records, credit card data, cell phone locations and movie ratings, a careful evaluation of the actual anonymity of the available data is needed. In addition, the definition of metadata varies between systems, which means that metadata in one system is considered data in others. Due to this, it is important to carefully define the types of data intelligence agencies and third-parties are allowed to collect and share.

We urge policymakers charged with evaluating the privacy-security dilemma to keep in mind the limitations of machine learning applied to mass-surveillance in the context of counterterrorism. With the increasing performance and accuracy of machine learning algorithms, we think that these techniques can assist law enforcement in finding patterns in properly fitted data sets, but at the same time they are far more intrusive. Considering the extreme sparsity of terror attacks and their versatility in planning and execution, we think that even with continuous progress in machine learning, training of the mass surveillance algorithms will be a challenge that should be evaluated carefully in the coming years against the consequences of big inaccuracies, and hence their effectiveness. Despite the fact that inaccuracies in current machine learning methods might entail opportunity costs related to human supervision, current technological hype might lead to over-adoption and consequent suboptimal resource allocation.

## Footnotes

1 Stephanie Bird (2013) points out that privacy and security are “at opposing ends of a continuum along which a balance point can and should be found, that is, that there is a theoretical point at which ‘enough security’ can be balanced against ‘enough privacy’”.
